# 7,8,9,10-Tetra­hydro­cyclo­hepta­[*b*]indol-6(5*H*)-one

**DOI:** 10.1107/S160053680802463X

**Published:** 2008-08-06

**Authors:** M. Sridharan, K. J. Rajendra Prasad, A. Thomas Gunaseelan, A. Thiruvalluvar, R. J. Butcher

**Affiliations:** a Department of Chemistry, Bharathiar University, Coimbatore 641 046, Tamilnadu, India; b PG Research Department of Physics, Rajah Serfoji Government College (Autonomous), Thanjavur 613 005, Tamilnadu, India; c Department of Chemistry, Howard University, 525 College Street NW, Washington, DC 20059, USA

## Abstract

In the title mol­ecule, C_13_H_13_NO, the dihedral angle between the benzene and pyrrole rings is 1.05 (5)°. The cyclo­heptene ring adopts a slightly distorted boat conformation. In the crystal structure, inter­molecular N—H⋯O hydrogen bonds form centrosymmetric dimers. A C—H⋯π inter­action, involving the benzene ring, is also found in the structure.

## Related literature

For a related structure, see: Sridharan *et al.* (2008 ▶).
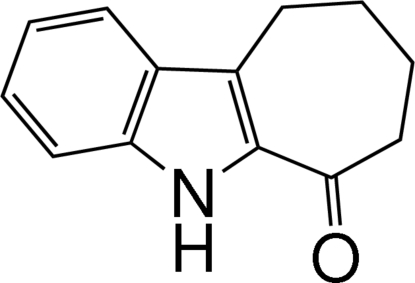


## Experimental

### 

#### Crystal data

C_13_H_13_NO
*M* *_r_* = 199.24
Monoclinic, 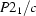

*a* = 14.0914 (4) Å
*b* = 8.0883 (2) Å
*c* = 9.2503 (3) Å
β = 108.937 (3)°
*V* = 997.24 (5) Å^3^
*Z* = 4
Mo *K*α radiation
μ = 0.08 mm^−1^
*T* = 200 (2) K
0.56 × 0.38 × 0.31 mm

#### Data collection

Oxford Diffraction Gemini R diffractometer
Absorption correction: multi-scan (*CrysAlis RED*; Oxford Diffraction, 2008 ▶) *T*_min_ = 0.985, *T*_max_ = 1.000 (expected range = 0.960–0.974)
15925 measured reflections
4127 independent reflections
3036 reflections with *I* > 2σ(*I*)
*R*_int_ = 0.023

#### Refinement

*R*[*F* ^2^ > 2σ(*F* ^2^)] = 0.052
*wR*(*F* ^2^) = 0.145
*S* = 1.01
4127 reflections
140 parameters
H atoms treated by a mixture of independent and constrained refinement
Δρ_max_ = 0.31 e Å^−3^
Δρ_min_ = −0.35 e Å^−3^

### 

Data collection: *CrysAlis CCD* (Oxford Diffraction, 2008 ▶); cell refinement: *CrysAlis RED* (Oxford Diffraction, 2008 ▶); data reduction: *CrysAlis RED*; program(s) used to solve structure: *SHELXS97* (Sheldrick, 2008 ▶); program(s) used to refine structure: *SHELXL97* (Sheldrick, 2008 ▶); molecular graphics: *ORTEP-3* (Farrugia, 1997 ▶); software used to prepare material for publication: *PLATON* (Spek, 2003 ▶).

## Supplementary Material

Crystal structure: contains datablocks global, I. DOI: 10.1107/S160053680802463X/wn2274sup1.cif

Structure factors: contains datablocks I. DOI: 10.1107/S160053680802463X/wn2274Isup2.hkl

Additional supplementary materials:  crystallographic information; 3D view; checkCIF report

## Figures and Tables

**Table 1:** Hydrogen-bond geometry (Å, °)

*D*—H⋯*A*	*D*—H	H⋯*A*	*D*⋯*A*	*D*—H⋯*A*
N5—H5⋯O6^i^	0.891 (16)	1.976 (16)	2.8188 (11)	157.3 (13)
C10—H10*A*⋯*Cg*^ii^	0.99	2.90	3.7087 (10)	139

Symmetry codes: (i) 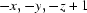; (ii) 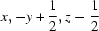. *Cg* is the centroid of the benzene ring.

**Fig. 1.**
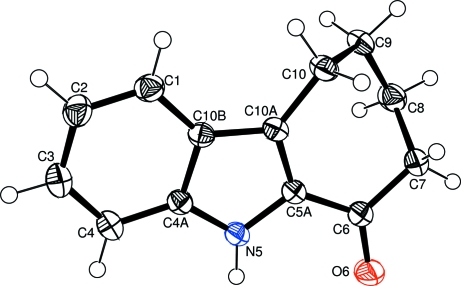
The molecular structure of the title compound, showing the atom-numbering scheme and displacement ellipsoids drawn at the 50% probability level. Hydrogen atoms are represented by spheres of arbitrary radius.

**Fig. 2.**
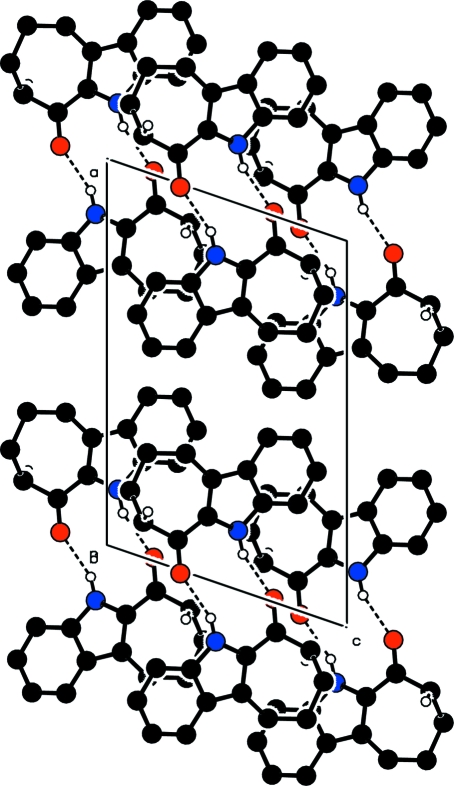
The molecular packing of the title compound, viewed down the b axis. Dashed lines indicate hydrogen bonds. H atoms not involved in hydrogen bonding have been omitted.

## supplementary crystallographic information

### Comment

Sridharan *et al.* (2008) have reported the crystal structure of 7,8,9,10-tetrahydro-2-methylcyclohepta[*b*]indol-6(5*H*)-one, in which the cycloheptene ring adopts a slightly distorted envelope conformation. The molecular structure of the title compound, with atomic numbering scheme, is shown in Fig. 1. The dihedral angle between the benzene ring and the pyrrole ring is 1.05 (5)°. The cycloheptene ring adopts a slightly distorted boat conformation. Intermolecular N5—H5···O6 (-*x*, -*y*, 1 - *z*) hydrogen bonds form centrosymmetric dimers in the crystal structure (Fig. 2). A C—H···π interaction, involving the benzene ring, is also found in the structure.

### Experimental

A solution of 2-(2-(4-phenyl)hydrazono)cycloheptanone (0.216 g, 0.001 mol) in a mixture of acetic acid (20 ml) and concentrated hydrochloric acid (5 ml) was refluxed on an oil bath pre-heated to 398–403 K for 2 h. The reaction was monitored by TLC. After the completion of reaction the contents were cooled and poured into ice water with stirring. The separated brown solid was filtered and purified by passing through a column of silica gel and eluting with petroleum ether-ethyl acetate (95:5 *v*/*v*) mixture to yield the title compound (0.129 g, 61%). The product thus obtained was recrystallized using ethanol.

### Refinement

The H atom bonded to N5 was located in a difference Fourier map and refined isotropically [N5—H5 = 0.891 (16) Å]. Other H atoms were positioned geometrically and allowed to ride on their parent atoms, with C—H = 0.95–0.99 Å and *U*_iso_(H) = 1.2*U*_eq_(parent atom).

### Crystal data

**Table:**

C_13_H_13_NO	*F*_000_ = 424
*M**_r_* = 199.24	*D*_x_ = 1.327 Mg m^−^^3^
Monoclinic, *P*2_1_/*c*	Melting point: 425(1) K
Hall symbol: -P 2ybc	Mo *K*α radiation λ = 0.71073 Å
*a* = 14.0914 (4) Å	Cell parameters from 7034 reflections
*b* = 8.0883 (2) Å	θ = 5.0–34.8º
*c* = 9.2503 (3) Å	µ = 0.08 mm^−^^1^
β = 108.937 (3)º	*T* = 200 (2) K
*V* = 997.24 (5) Å^3^	Chunk, pale-yellow
*Z* = 4	0.56 × 0.38 × 0.31 mm

### Data collection

**Table:**

Oxford Diffraction R Gemini diffractometer	4127 independent reflections
Radiation source: fine-focus sealed tube	3036 reflections with *I* > 2σ(*I*)
Monochromator: graphite	*R*_int_ = 0.023
Detector resolution: 10.5081 pixels mm^-1^	θ_max_ = 34.9º
*T* = 200(2) K	θ_min_ = 5.0º
φ and ω scans	*h* = −22→22
Absorption correction: multi-scan(CrysAlis RED; Oxford Diffraction, 2008)	*k* = −12→12
*T*_min_ = 0.985, *T*_max_ = 1.000	*l* = −11→14
15925 measured reflections	

### Refinement

**Table:**

Refinement on *F*^2^	Secondary atom site location: difference Fourier map
Least-squares matrix: full	Hydrogen site location: inferred from neighbouring sites
*R*[*F*^2^ > 2σ(*F*^2^)] = 0.052	H atoms treated by a mixture of independent and constrained refinement
*wR*(*F*^2^) = 0.145	*w* = 1/[σ^2^(*F*_o_^2^) + (0.097*P*)^2^] where *P* = (*F*_o_^2^ + 2*F*_c_^2^)/3
*S* = 1.01	(Δ/σ)_max_ = 0.001
4127 reflections	Δρ_max_ = 0.31 e Å^−^^3^
140 parameters	Δρ_min_ = −0.35 e Å^−^^3^
Primary atom site location: structure-invariant direct methods	Extinction correction: none

### Special details

**Table:**

Geometry. Bond distances, angles *etc*. have been calculated using the rounded fractional coordinates. All su's are estimated from the variances of the (full) variance-covariance matrix. The cell e.s.d.'s are taken into account in the estimation of distances, angles and torsion angles
Refinement. Refinement of *F*^2^ against ALL reflections. The weighted *R*-factor *wR* and goodness of fit *S* are based on *F*^2^, conventional *R*-factors *R* are based on *F*, with *F* set to zero for negative *F*^2^. The threshold expression of *F*^2^ > σ(*F*^2^) is used only for calculating *R*-factors(gt) *etc*. and is not relevant to the choice of reflections for refinement. *R*-factors based on *F*^2^ are statistically about twice as large as those based on *F*, and *R*- factors based on ALL data will be even larger.

### Fractional atomic coordinates and isotropic or equivalent isotropic displacement parameters (Å^2^)

**Table:**

	*x*	*y*	*z*	*U*_iso_*/*U*_eq_	
O6	−0.00855 (5)	0.08244 (11)	0.30591 (9)	0.0440 (2)	
N5	0.15340 (5)	−0.04196 (9)	0.54651 (8)	0.0256 (2)	
C1	0.42047 (7)	−0.01523 (11)	0.66953 (11)	0.0298 (2)	
C2	0.44834 (7)	−0.08626 (12)	0.81195 (11)	0.0343 (3)	
C3	0.37614 (7)	−0.14708 (12)	0.87403 (11)	0.0332 (3)	
C4	0.27498 (7)	−0.13868 (10)	0.79473 (10)	0.0284 (2)	
C4A	0.24673 (6)	−0.06767 (10)	0.64909 (9)	0.0232 (2)	
C5A	0.16322 (6)	0.03677 (10)	0.41960 (10)	0.0242 (2)	
C6	0.07608 (6)	0.08971 (11)	0.29509 (10)	0.0280 (2)	
C7	0.09234 (7)	0.15126 (12)	0.15191 (11)	0.0321 (3)	
C8	0.16098 (8)	0.03791 (13)	0.09678 (11)	0.0349 (3)	
C9	0.27357 (7)	0.06922 (12)	0.16778 (11)	0.0312 (2)	
C10	0.30600 (6)	0.14407 (11)	0.32855 (10)	0.0293 (2)	
C10A	0.26382 (6)	0.05963 (10)	0.43769 (10)	0.0235 (2)	
C10B	0.31807 (6)	−0.00584 (10)	0.58454 (10)	0.0235 (2)	
H1	0.46963	0.02683	0.62920	0.0357*	
H2	0.51753	−0.09456	0.86970	0.0412*	
H3	0.39782	−0.19511	0.97311	0.0398*	
H4	0.22650	−0.17934	0.83711	0.0340*	
H5	0.0970 (11)	−0.0616 (16)	0.5673 (16)	0.049 (4)*	
H7A	0.02662	0.16053	0.07022	0.0385*	
H7B	0.12233	0.26314	0.17084	0.0385*	
H8A	0.14277	0.04968	−0.01546	0.0419*	
H8B	0.14723	−0.07800	0.11811	0.0419*	
H9A	0.30934	−0.03693	0.17193	0.0375*	
H9B	0.29503	0.14442	0.09981	0.0375*	
H10A	0.28542	0.26165	0.32052	0.0351*	
H10B	0.38014	0.14072	0.37110	0.0351*	

### Atomic displacement parameters (Å^2^)

**Table:**

	*U*^11^	*U*^22^	*U*^33^	*U*^12^	*U*^13^	*U*^23^
O6	0.0248 (3)	0.0711 (5)	0.0371 (4)	0.0026 (3)	0.0116 (3)	0.0130 (4)
N5	0.0247 (3)	0.0298 (3)	0.0240 (3)	−0.0015 (3)	0.0102 (3)	0.0031 (3)
C1	0.0252 (4)	0.0355 (4)	0.0284 (4)	−0.0014 (3)	0.0084 (3)	−0.0035 (3)
C2	0.0296 (4)	0.0410 (5)	0.0283 (4)	0.0012 (4)	0.0038 (3)	−0.0013 (4)
C3	0.0391 (5)	0.0331 (4)	0.0239 (4)	0.0006 (3)	0.0055 (3)	0.0021 (3)
C4	0.0358 (4)	0.0266 (4)	0.0231 (4)	−0.0022 (3)	0.0101 (3)	0.0008 (3)
C4A	0.0262 (3)	0.0217 (3)	0.0224 (4)	−0.0004 (3)	0.0088 (3)	−0.0016 (3)
C5A	0.0252 (4)	0.0256 (3)	0.0229 (4)	−0.0003 (3)	0.0094 (3)	0.0017 (3)
C6	0.0262 (4)	0.0316 (4)	0.0268 (4)	0.0006 (3)	0.0096 (3)	0.0035 (3)
C7	0.0308 (4)	0.0386 (5)	0.0270 (4)	0.0031 (3)	0.0097 (3)	0.0091 (3)
C8	0.0370 (5)	0.0436 (5)	0.0250 (4)	−0.0005 (4)	0.0113 (4)	−0.0009 (4)
C9	0.0358 (4)	0.0367 (4)	0.0261 (4)	0.0016 (3)	0.0168 (3)	0.0049 (3)
C10	0.0295 (4)	0.0326 (4)	0.0289 (4)	−0.0038 (3)	0.0139 (3)	0.0023 (3)
C10A	0.0257 (4)	0.0240 (3)	0.0230 (4)	−0.0010 (3)	0.0109 (3)	−0.0006 (3)
C10B	0.0247 (3)	0.0238 (3)	0.0226 (4)	−0.0007 (3)	0.0087 (3)	−0.0024 (3)

### Geometric parameters (Å, °)

**Table:**

O6—C6	1.2301 (12)	C9—C10	1.5316 (13)
N5—C4A	1.3650 (11)	C10—C10A	1.4928 (12)
N5—C5A	1.3814 (11)	C10A—C10B	1.4269 (12)
N5—H5	0.891 (16)	C1—H1	0.9500
C1—C10B	1.4035 (14)	C2—H2	0.9500
C1—C2	1.3726 (14)	C3—H3	0.9500
C2—C3	1.4093 (14)	C4—H4	0.9500
C3—C4	1.3770 (14)	C7—H7A	0.9900
C4—C4A	1.3983 (12)	C7—H7B	0.9900
C4A—C10B	1.4166 (12)	C8—H8A	0.9900
C5A—C6	1.4491 (12)	C8—H8B	0.9900
C5A—C10A	1.3849 (13)	C9—H9A	0.9900
C6—C7	1.5005 (13)	C9—H9B	0.9900
C7—C8	1.5350 (15)	C10—H10A	0.9900
C8—C9	1.5285 (15)	C10—H10B	0.9900
			
O6···N5	2.8066 (11)	H2···H9B^vii^	2.6000
O6···N5^i^	2.8188 (11)	H3···C1^v^	2.9200
O6···H5	2.661 (14)	H3···C10B^v^	2.9900
O6···H5^i^	1.976 (16)	H5···O6	2.661 (14)
N5···O6	2.8066 (11)	H5···O6^i^	1.976 (16)
N5···O6^i^	2.8188 (11)	H5···H7B^ii^	2.5800
N5···H7B^ii^	2.6300	H7A···C8^vi^	3.0500
C2···C2^iii^	3.5893 (14)	H7A···H8B^vi^	2.5900
C1···H10B	2.9200	H7B···C10	2.7000
C1···H3^iv^	2.9200	H7B···C10A	3.1000
C3···H2^iii^	3.0600	H7B···H10A	2.2700
C4···H8B^v^	3.0400	H7B···N5^viii^	2.6300
C4···H9A^v^	2.9600	H7B···C4A^viii^	3.0700
C4···H10A^ii^	3.0600	H7B···C5A^viii^	3.0400
C4A···H7B^ii^	3.0700	H7B···H5^viii^	2.5800
C4A···H10A^ii^	2.8900	H8B···C5A	2.8800
C5A···H8B	2.8800	H8B···C10A	3.0900
C5A···H7B^ii^	3.0400	H8B···H7A^vi^	2.5900
C7···H10A	2.8100	H8B···C4^iv^	3.0400
C8···H7A^vi^	3.0500	H9A···C4^iv^	2.9600
C9···H2^vii^	3.0800	H9B···H2^vii^	2.6000
C10···H7B	2.7000	H9B···C10^viii^	3.0800
C10···H9B^ii^	3.0800	H9B···C10A^viii^	2.7800
C10A···H7B	3.1000	H9B···C10B^viii^	2.9500
C10A···H8B	3.0900	H10A···C7	2.8100
C10A···H9B^ii^	2.7800	H10A···H7B	2.2700
C10B···H3^iv^	2.9900	H10A···C4^viii^	3.0600
C10B···H9B^ii^	2.9500	H10A···C4A^viii^	2.8900
C10B···H10A^ii^	3.0900	H10A···C10B^viii^	3.0900
H1···H10B	2.4900	H10B···C1	2.9200
H1···H10B^vii^	2.5100	H10B···H1	2.4900
H2···C3^iii^	3.0600	H10B···H1^vii^	2.5100
H2···C9^vii^	3.0800		
			
C4A—N5—C5A	108.75 (7)	C10B—C1—H1	121.00
C4A—N5—H5	123.3 (9)	C1—C2—H2	119.00
C5A—N5—H5	127.5 (9)	C3—C2—H2	119.00
C2—C1—C10B	118.83 (9)	C2—C3—H3	119.00
C1—C2—C3	121.18 (9)	C4—C3—H3	119.00
C2—C3—C4	121.68 (9)	C3—C4—H4	121.00
C3—C4—C4A	117.09 (9)	C4A—C4—H4	121.00
N5—C4A—C4	129.88 (8)	C6—C7—H7A	109.00
N5—C4A—C10B	107.98 (7)	C6—C7—H7B	109.00
C4—C4A—C10B	122.14 (8)	C8—C7—H7A	109.00
N5—C5A—C6	121.26 (8)	C8—C7—H7B	109.00
C6—C5A—C10A	128.81 (8)	H7A—C7—H7B	108.00
N5—C5A—C10A	109.86 (8)	C7—C8—H8A	108.00
O6—C6—C7	121.16 (8)	C7—C8—H8B	108.00
C5A—C6—C7	117.83 (8)	C9—C8—H8A	108.00
O6—C6—C5A	121.00 (8)	C9—C8—H8B	108.00
C6—C7—C8	112.85 (8)	H8A—C8—H8B	107.00
C7—C8—C9	115.87 (8)	C8—C9—H9A	109.00
C8—C9—C10	115.07 (8)	C8—C9—H9B	108.00
C9—C10—C10A	114.65 (8)	C10—C9—H9A	109.00
C5A—C10A—C10B	106.06 (8)	C10—C9—H9B	109.00
C10—C10A—C10B	127.28 (8)	H9A—C9—H9B	108.00
C5A—C10A—C10	126.58 (8)	C9—C10—H10A	109.00
C4A—C10B—C10A	107.34 (8)	C9—C10—H10B	109.00
C1—C10B—C4A	119.08 (8)	C10A—C10—H10A	109.00
C1—C10B—C10A	133.56 (8)	C10A—C10—H10B	109.00
C2—C1—H1	121.00	H10A—C10—H10B	108.00
			
C5A—N5—C4A—C4	178.26 (8)	C10A—C5A—C6—O6	−168.79 (9)
C5A—N5—C4A—C10B	−0.94 (9)	C10A—C5A—C6—C7	12.09 (14)
C4A—N5—C5A—C6	−176.03 (8)	N5—C5A—C10A—C10	−178.13 (8)
C4A—N5—C5A—C10A	1.30 (10)	N5—C5A—C10A—C10B	−1.11 (9)
C2—C1—C10B—C10A	179.18 (9)	C6—C5A—C10A—C10	−1.05 (15)
C2—C1—C10B—C4A	0.89 (13)	C6—C5A—C10A—C10B	175.96 (8)
C10B—C1—C2—C3	−0.89 (14)	O6—C6—C7—C8	−132.19 (10)
C1—C2—C3—C4	0.30 (15)	C5A—C6—C7—C8	46.93 (11)
C2—C3—C4—C4A	0.29 (13)	C6—C7—C8—C9	−85.91 (11)
C3—C4—C4A—C10B	−0.27 (12)	C7—C8—C9—C10	27.65 (12)
C3—C4—C4A—N5	−179.36 (9)	C8—C9—C10—C10A	48.32 (11)
N5—C4A—C10B—C1	178.95 (8)	C9—C10—C10A—C5A	−57.72 (12)
C4—C4A—C10B—C10A	−179.02 (8)	C9—C10—C10A—C10B	125.89 (9)
N5—C4A—C10B—C10A	0.25 (9)	C5A—C10A—C10B—C1	−177.91 (9)
C4—C4A—C10B—C1	−0.32 (12)	C5A—C10A—C10B—C4A	0.53 (9)
N5—C5A—C6—O6	7.99 (13)	C10—C10A—C10B—C1	−0.92 (16)
N5—C5A—C6—C7	−171.13 (8)	C10—C10A—C10B—C4A	177.51 (8)

Symmetry codes: (i) −*x*, −*y*, −*z*+1; (ii) *x*, −*y*+1/2, *z*+1/2; (iii) −*x*+1, −*y*, −*z*+2; (iv) *x*, −*y*−1/2, *z*−1/2; (v) *x*, −*y*−1/2, *z*+1/2; (vi) −*x*, −*y*, −*z*; (vii) −*x*+1, −*y*, −*z*+1; (viii) *x*, −*y*+1/2, *z*−1/2.

### Hydrogen-bond geometry (Å, °)

**Table:**

*D*—H···*A*	*D*—H	H···*A*	*D*···*A*	*D*—H···*A*
N5—H5···O6^i^	0.891 (16)	1.976 (16)	2.8188 (11)	157.3 (13)
C10—H10A···Cg^viii^	0.99	2.90	3.7087 (10)	139

Symmetry codes: (i) −*x*, −*y*, −*z*+1; (viii) *x*, −*y*+1/2, *z*−1/2.
